# Study of the Synchrotron Photoionization Oxidation of Alpha-Angelica Lactone (AAL) Initiated by O(^3^P) at 298, 550, and 700 K

**DOI:** 10.3390/molecules26134070

**Published:** 2021-07-03

**Authors:** Golbon Rezaei, Giovanni Meloni

**Affiliations:** 1 Department of Chemistry, University of San Francisco, San Francisco, CA 94117, USA; grezaei@dons.usfca.edu; 2 Department of Physical and Chemical Sciences, Università degli Studi de L’Aquila, 67100 L’Aquila, Italy

**Keywords:** biofuel, oxidation, reaction pathways, synchrotron photoionization, multiplexed mass spectrometry

## Abstract

In recent years, biofuels have been receiving significant attention because of their potential for decreasing carbon emissions and providing a long-term renewable solution to unsustainable fossil fuels. Currently, lactones are some of the alternatives being produced. Many lactones occur in a range of natural substances and have many advantages over bioethanol. In this study, the oxidation of alpha-angelica lactone initiated by ground-state atomic oxygen, O(^3^P), was studied at 298, 550, and 700 K using synchrotron radiation coupled with multiplexed photoionization mass spectrometry at the Lawrence Berkeley National Lab (LBNL). Photoionization spectra and kinetic time traces were measured to identify the primary products. Ketene, acetaldehyde, methyl vinyl ketone, methylglyoxal, dimethyl glyoxal, and 5-methyl-2,4-furandione were characterized as major reaction products, with ketene being the most abundant at all three temperatures. Possible reaction pathways for the formation of the observed primary products were computed using the CBS–QB3 composite method.

## 1. Introduction

During the last century, fossil fuels have been used as the main source of energy in the world [1]. Natural sources, such as the burning of forests and volcanoes, human activities including the combustion of fossil fuels for transportation and power generation, and agriculture, release billions of tons of pollutants into the atmosphere each year [2,3]. Fossil fuel combustion increases in the atmosphere the levels of dangerous contaminants, such as sulfur dioxide (SO_2_), nitrogen dioxide (NO_2_), carbon monoxide (CO), ground-level ozone, and particulate matter (PM), which cause environmental damage and are dangerous for human health [1,4].

Fossil fuel sources are diminishing, while the global demand for energy is growing. Thus, investments in finding alternative fuels, which would be more efficient, sustainable, and environmentally friendly, are increasing [5,6,7,8,9]. In recent years, carbon-based bio-derived compounds have become the focus of research aimed at finding an immediate alternative to fossil fuels [10]. Specifically, biofuels are an interesting research area because of their potential to decrease carbon emissions and provide a long-term renewable solution to unsustainable fossil fuels [7,11]. Other advantages of biofuels as a more attractive alternative are their accessible source and their relative ease of processing [12]. Different biofuel alternatives are already produced commercially on the industrial scale for public use [13].

In general, biofuels are divided into three categories: first, second, and third generation. The difference is the source from which they are produced. First-generation biofuels are derived from food crops, whereas second-generation biofuels are obtained from biomass, and third-generation ones comes from algae [14]. Second-generation biofuels are obtained from non-edible biomass, such as waste vegetable oil, municipal solid waste, or lignocellulosic biomass as feedstock [15]. This generation of biofuels, produced from lignocellulosic materials (LCMs), is presumed to decrease greenhouse gas emissions more than the first generation of biofuels [16]. LCM is one of the most plentiful forms of renewable carbon on earth [17]. It consists of an aromatic polymer (lignin) and bonded carbohydrate polymers (cellulose and hemicellulose), which are converted into valuable fuels through different reactions [17,18,19,20].

Currently, esters, ethers, alcohols, furans, and lactones are some of the alternatives being produced [5,20,21,22,23,24,25,26,27,28,29,30,31,32]. Many lactones occur in a range of natural substances and have many advantages over bioethanol, which is one of the world’s most employed renewable sources [33]. The combustion of saturated hydrocarbons [34,35,36,37,38,39], unlike the combustion of lactonic biofuels, has been studied widely. Lactones are miscible with water, but they are more easily isolated through distillation than ethanol. Besides, lactones are stable under neutral conditions and are not oxidized in the air. They are safe to store and move because of their high boiling point and low melting point [33].

Angelica lactones (α, β, and γ angelica lactone) are classified as butenolides, which are lactones with four carbon atoms in their heterocyclic structure. These five-membered heterocycles are essential synthons that are found in several natural products, specifically, in compounds with biological activities [40]. Alpha-angelica lactone (T_b_ = 167–170 °C) and beta-angelica lactone (T_b_ = 208–209 °C) are volatile species. Alpha-angelica lactone (AAL) has an odor similar to that of coconut, chocolate, or vanilla and it is traditionally used in perfumes. In the past, an angelica root extract containing angelica lactones has been used as a tobacco additive, imparting a smoothing, caramel smoke taste. AAL has been found in *Angelica* genus plants, raisins, white bread, soybeans, and licorice [41]. Recently, AAL was defined as a new platform molecule, which can be created from the lignocellulosic material levulinic acid (LA) with a 95% yield, making its synthesis easily achievable [42].

This investigation is focused on the characterization of AAL oxidation initial steps at 298, 550, and 700 K initiated by O(^3^P), using synchrotron photoionization multiplexed mass spectrometry. This low-temperature region is particularly important for understanding and predicting the autoignition behavior of fuels [39,43], which is relevant in new advanced engines, based, for instance on HCCI (homogeneous-charge compression ignition). The primary products were identified through their photoionization spectra, and their formation was explained via computation of the potential energy surface using the CBS–QB3 composite model.

## 2. Results and Discussion

**Scheme 1:**
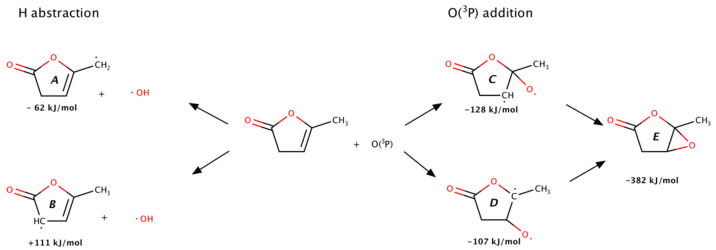
Two possible triplet diradicals formed from the oxygen addition pathway (C and D) and the singlet epoxide (E).

The oxidation of AAL initiated by O(^3^P) has two possible pathways: oxygen addition to the unsaturated carbons and H abstraction by O(^3^P). Scheme 1 presents the pathways of the mentioned reaction with the relative energies of two different triplet diradicals (C and D), one singlet epoxide molecule (E), and two doublet radicals from hydrogen abstraction (A and B). The most energetically favorable pathway is O(^3^P) addition to the most substituted carbon of the double bond, whereas the least energetically favorable pathway is hydrogen abstraction from the ring, due to the stronger C(sp^2^)–H bond. In general, H abstraction by O(^3^P) channels is kinetically hindered because of the presence of large barriers [44,45]. As seen in Scheme 1, the diradicals C and D formed from adding the ground-state oxygen atom to the unsaturated carbons. The reaction’s enthalpies (Δ_r_H) are presented at 0 K. The enthalpy change for the triplet diradical C resulted to be −128 kJ mol^−1^, and that for the triplet diradical D −107 kJ mol^−1^. These two triplet diradicals underwent intersystem crossing (ISC) to form the singlet epoxide (E) at −382 kJ mol^−1^ which led to the several products observed in this work.

The AAL + O(^3^P) reactions were carried out at three different temperatures. At 298 K, the measurements were performed from 8.4 to 11 eV, at 550 and 700 K in the 8.2–11 eV and 9.4–11 eV ranges, respectively.

### 2.1. Product Identification

**Figure 1:**
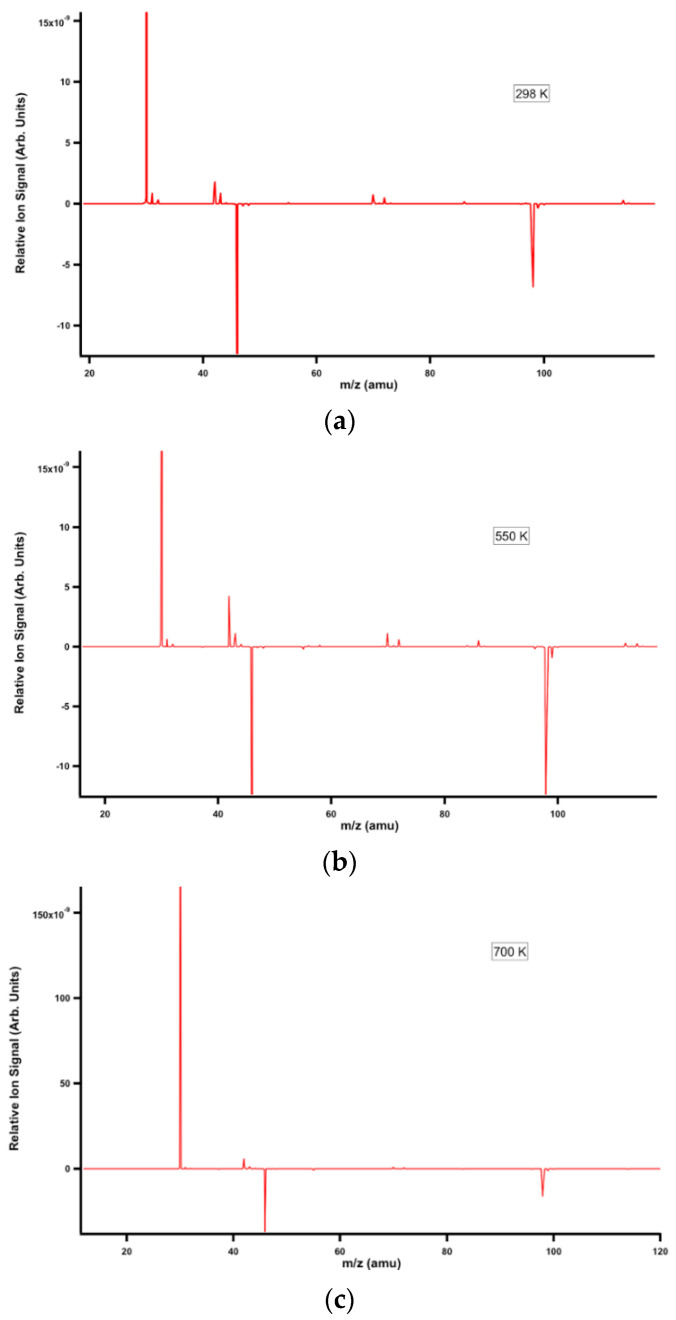
Photoionization mass spectra at (**a**) 298 K, (**b**) 550 K, and (**c**) 700 K. These mass spectra were collected over the photon energy range of 8.4–11.0 eV at 298 K, 8.2–11 eV at 550 K, and 9.4–11 eV at 700 K.

**Figure 2:**
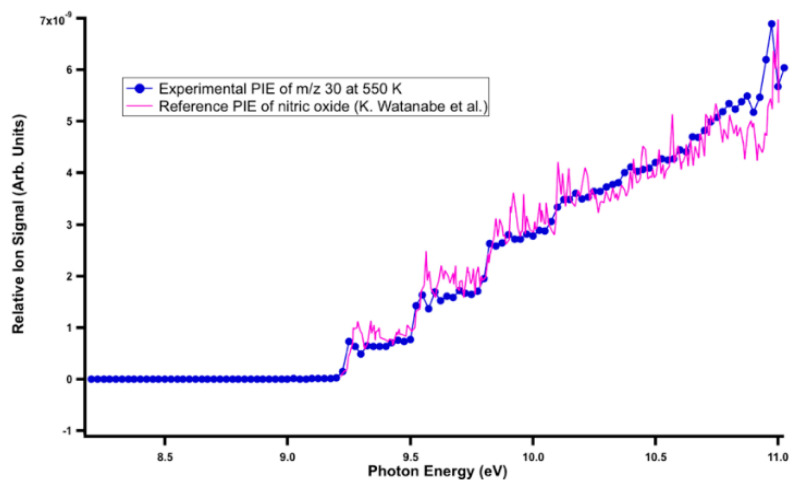
Reference PI spectrum of NO (blue line) superimposed onto the experimental PI curve at *m*/*z* 30 at 550 K.

In the AAL + O(^3^P) reaction, the primary products formed at *m*/*z* 42, 44, 70, 72, and 114 at 298 and 550 K, in addition to *m*/*z* 86 at 550 K, *m*/*z* 42, 44, 70, 72, and 86 at 700 K. The photoionization mass spectra at the three temperatures are presented in Figure 1. The reactant showed a negative ion signal corresponding to a depleting species, and the products presented positive ion signals corresponding to species formation. By photolyzing NO_2_ to produce O(^3^P), NO formed at *m*/*z* 30. Its experimental photoionization (PI) spectrum matched with the literature PI spectrum of NO [46]. Figure 2 shows the PI curve of *m*/*z* 30 at 550 K.

**Figure 3:**
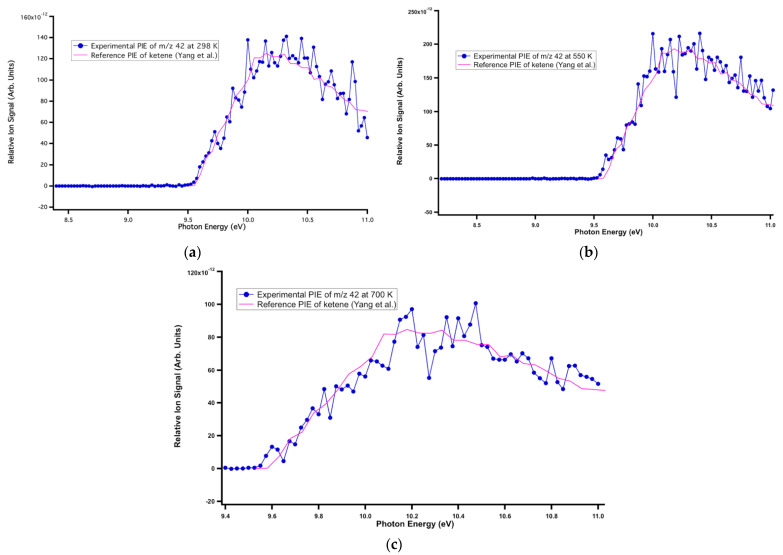
Literature PI spectra of ketene superimposed onto the experimental PI spectra of *m*/*z* 42 at (**a**) 298 K (**b**) 550 K, and (**c**) 700 K.

**Figure 4:**
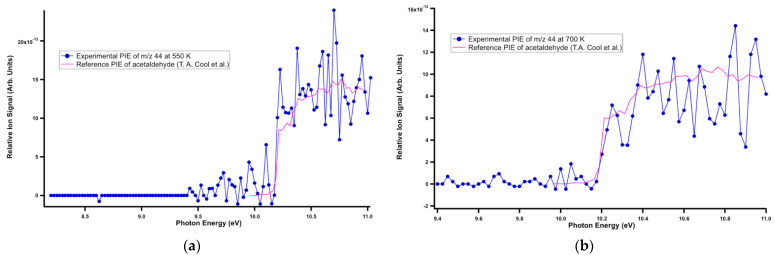
Reference PI spectrum of acetaldehyde superimposed onto the experimental PI spectrum at *m*/*z* 44 at (**a**) 550 K and (**b**) 700 K.

**Figure 5:**
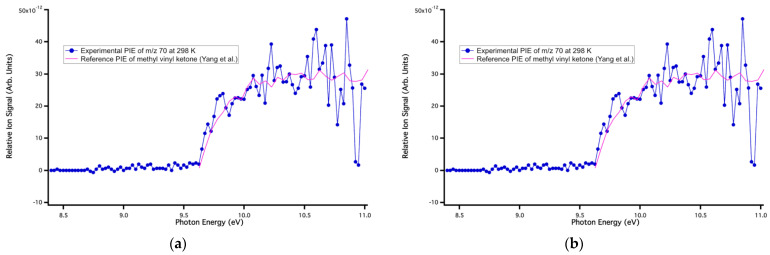
Reference PI spectrum of methyl vinyl ketone superimposed onto the experimental PI spectrum at *m*/*z* 70 at (**a**) 298 K and (**b**) 550 K.

**Figure 6:**
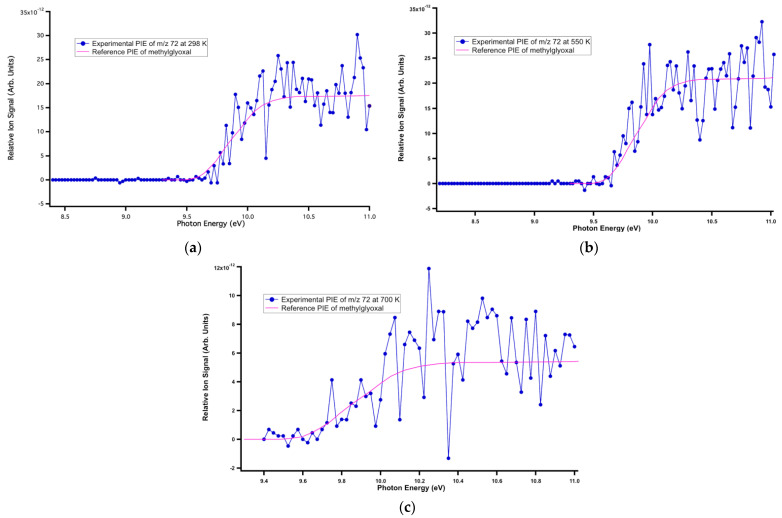
Franck–Condon simulated PI spectrum of methylglyoxal superimposed onto the experimental PI spectrum at *m*/*z* 72 at (**a**) 298 K, (**b**) 550 K, and (**c**) 700 K.

**Figure 7:**
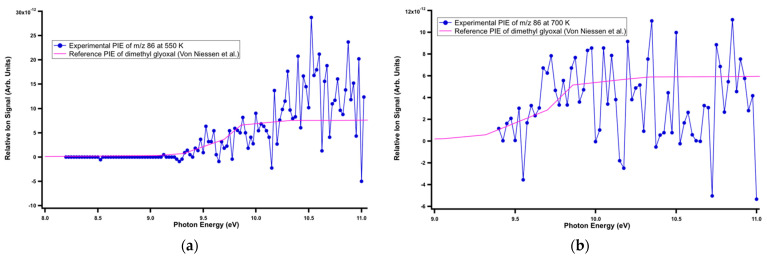
Reference PI spectrum of dimethyl glyoxal superimposed onto the experimental PI spectrum at *m*/*z* 86 at (**a**) 550 K and (**b**) 700 K.

**Figure 8:**
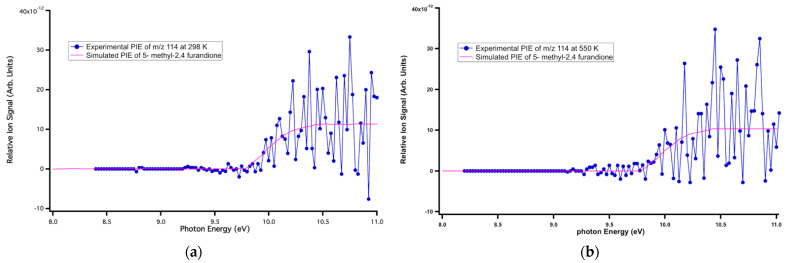
Franck–Condon simulated PI spectrum of 5-methyl-2,4-furandione superimposed onto the experimental PI spectrum at *m*/*z* 114 at (**a**) 550 K and (**b**) 700 K.

The signal at *m*/*z* 42 was assigned to ketene. Its literature PI spectrum reported by Goulay et al. [47] matched well with the experimental data at *m*/*z* 42 at all temperatures (Figure 3). The measured ionization energy of 9.56 ± 0.05 eV agreed with the reference adiabatic ionization energy (AIE) of 9.60 ± 0.01 eV [48]. The signal at *m*/*z* 44 was assigned to acetaldehyde. The experimental PI spectrum of acetaldehyde matched well with the literature PI curve [38] at all temperatures, as observed in Figure 4, and the measured ionization energy of 10.18 ± 0.05 eV was in good agreement with the literature value of 10.20 ± 0.25 eV [38]. As shown in Figure 5, the reference PI spectra of methyl vinyl ketone reported by Yang et al. [48] agree well with the experimental PI spectra at *m*/*z* 70 at all three temperatures. The ion signal starting at ~10.6 eV showed an artificial drop most likely due to an over-background subtraction of a very low signal-to-noise-ratio. The experimental ionization energy of 9.60 ± 0.05 eV matched the reference AIE of 9.62 ± 0.01 eV [47]. The product at *m*/*z* 72 was observed at all three temperatures and assigned to methylglyoxal based on the experimental ionization onset’s good agreement at 9.56 ± 0.01 eV with the literature AIE of 9.60 ± 0.05 eV [49] (Figure 6). The experimental ionization energy of 9.11 ± 0.01 eV matched the literature PI curve of dimethyl glyoxal with an AIE of 9.06 ± 0.05 eV, but because of the low signal-to-noise ratio, its characterization was tentative (Figure 7). The last identified product at 298 and 550 K was at *m*/*z* 114 and corresponded to 5-methyl-2,4 furandione. No spectra are available in the literature for this species, and an FC simulation was performed and agreed with the experimental data (Figure 8). The higher portion of this spectrum presented a low signal-to-noise ratio that affected the determination of its branching fraction.

### 2.2. Branching Fractions

**Figure 9:**
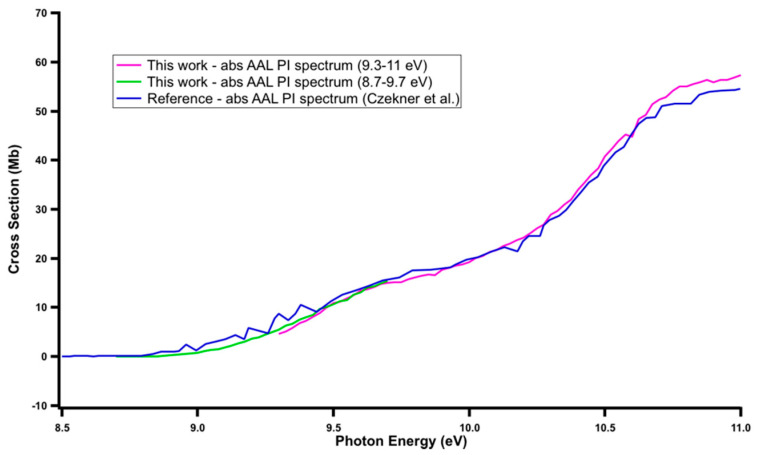
Absolute literature PI spectrum of AAL (blue line) superimposed onto the experimental PI curve (pink and green line) collected at 298 K.

Thorstad et al. [50] reported that the AAL ionization energy is 9.62 ± 0.05 eV using electron ionization techniques. Figure 9 shows the superimposed absolute AAL PI curve from the literature [51] onto the experimental data, with an adiabatic ionization energy of 8.97 ± 0.05 eV, which is much lower than the previously reported value. Thorstad et al. [50] also reported that AAL does not have dissociative fragments at lower ionization energies, unlike other lactones, because there are no hydrogens on adjacent carbon atoms to create a stable resonance structure.

It is essential to know the reactant and each primary product’s concentration to calculate the branching fractions. The branching fractions of all primary products formed at the three examined temperatures, obtained using Equation (1), are shown in Table 1. The signal that was used to compute the branching fractions at the three temperatures was averaged for 250 laser shots at each energy step. For convenience, the PI spectra of each primary product were compared at the same photon energy of 11 eV. The branching fractions’ uncertainties were computed by the propagation errors of variables in Equation (1) and are shown in Table 1. The uncertainties of the mass discrimination and photoionization cross-sections were taken from the literature; the uncertainties of ion signal were obtained by taking the difference between the measured upper or lower ion signal value at 11 eV and the reference spectrum (simulation or literature) of a specific species at 11 eV.

In order to proceed with a proper quantification of the observed products, the branching fractions were carbon atom-balanced. This means that for our reactant AAL, the total branching fractions summed to 500% because of the presence of five carbon atoms. Therefore, each single branching fraction was multiplied by the C atom number present in the specific product and divided by 5 to report the total branching fractions to 100%. At 298 and 550 K, five products, i.e., ketene, acetaldehyde, methyl vinyl ketone, methylglyoxal, and 5-methyl-2,4-furandione were observed, and the total branching fraction was 101 ± 19.9%. The largest contribution was from ketene at this temperature, 47.2 ± 14.6%. At 550 K, the same primary products plus dimethyl glyoxal were obtained; the total branching fraction was 87.3 ± 18.1%. At 700 K, five products, i.e., ketene, acetaldehyde, methyl vinyl ketone, methylglyoxal, and dimethyl glyoxal were produced, and the total branching fraction was 83.4 ± 16.3%.

The branching fractions of ketene, acetaldehyde, and methyl vinyl ketone were computed using the literature photoionization cross sections at 11 eV of 16 ± 1.6 [48], 8.5 ± 2.1 [38], and 11 ± 1.1 Mb [48], respectively. The photoionization cross section of methylglyoxal, dimethyl glyoxal, and 5-methyl-2,4-furandione are not reported in the literature; thus, the semi-empirical additivity method [52] was used to calculate these values. Specifically, the photoionization cross section at 11 eV of methylglyoxal, CH_3_C(O)C(O)H, in which there are two C–C and two C=O bonds, can be approximated as the sum of the literature photoionization cross sections at 11 eV of two acetaldehyde molecules, CH_3_C(O)H, (8.5 ± 2.1 Mb) [38], in which there are one C–C and one C=O bond, yielding σ(methylglyoxal) = 17 ± 3.0 Mb. The photoionization cross section at 11 eV of dimethyl glyoxal, CH_3_C(O)C(O)CH_3_, in which there are three C–C and two C=O bonds, can be approximated as the sum of the literature photoionization cross sections of acetone (10 ± 2.5 Mb) [53], CH_3_C(O)CH_3_, in which there are two C–C and one C=O bonds, and acetaldehyde yielding σ(dimethyl glyoxal) = 18.5 ± 3.3 Mb. Finally, for 5-methyl-2,4-furandione, we added 13 Mb [52] to the photoionization cross section of AAL for the additional C=O bond, yielding σ(5-methyl-2,4-furandione) = 67 ± 17 Mb. With respect to the total yield, ketene contributed around (47 ± 15)% at 298 K, (40 ± 13)% at 550 K and (40 ± 12)% at 700 K, followed by methyl vinyl ketone (35 ± 13)% at 298 K, (27 ± 11)% at 550 K, and (20 ± 8)% at 700 K. Methylglyoxal was the third most abundant product at 298 K, (11 ± 4)%, whereas acetaldehyde was the third most abundant product at both 550 K, (9 ± 4)%, and 700 K, (16 ± 7)%.

### 2.3. Reaction Pathways for the Primary Products

**Figure 10:**
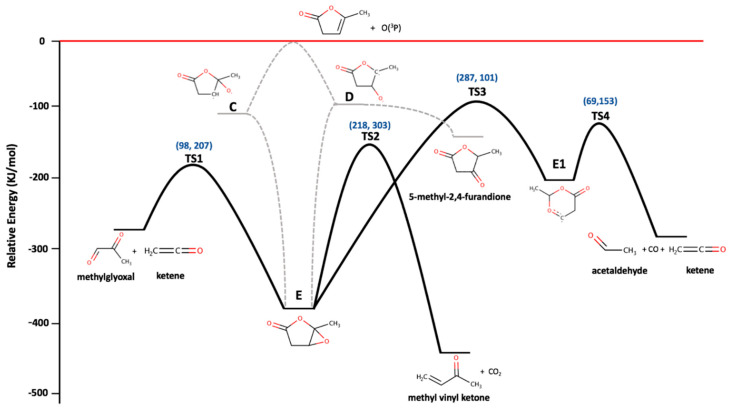
Potential energy surface diagram at 0 K calculated using the CBS–QB3 composite model representing the formation of primary products in the AAL + O(^3^P) reaction. The red line shows the reference zero level, i.e., AAL + O(^3^P). The dashed line from the triplet diradicals shows the intersystem crossing to the singlet surface.

In these experiments, the only observed pathway yielding the formation of the products was O(^3^P) addition to the unsaturated carbons. The hydrogen abstraction pathway by atomic oxygen is kinetically unfavorable because it proceeds via an energy barrier [44,45]. All primary products originated from intersystem crossing from the triplet to the singlet surface, i.e., from the initial diradicals C and D to the epoxide E and from the diradical D to dimethyl glyoxal and 5-methyl-2,4-furandione (Scheme 1). The values in parentheses represent the energy barriers from both directions of the transition state (TS). The AAL + O(^3^P) reaction was used as a reference point (the zero-energy level) to calculate the identified primary products’ energy. In the potential energy surface diagram (Figure 10), any molecular species and energetic barrier above the red line is kinetically unfavorable. The AAL oxidation reaction began with two possible triplet diradicals (C and D), which through intersystem crossing, formed the singlet epoxide (E). This species was not observed, because its cation is computed to be unbound using the CBS–QB3 composite model. From the epoxide, four products formed, which were ketene, acetaldehyde, methyl vinyl ketone, and methylglyoxal. Figure 10 shows that the pathways to the left of epoxide E leads to ketene and methylglyoxal, and the pathways to the right leads to the formation of acetaldehyde, methyl vinyl ketone, and 5-methyl-2,4-furandione.

Epoxide E went through intermediates E1 via hydrogen transfer from the β-carbon to the γ-carbon to form acetaldehyde and ketene. The first energy barrier TS3 to form E1 was the highest barrier in the computed potential energy surface, 287 kJ mol^−1^. The reaction enthalpy to yield acetaldehyde and ketene with CO from AAL + O(^3^P) was −281 kJ mol^−1^. The formation of methyl vinyl ketone occurred in a single step, with a computed barrier of 218 kJ mol^−1^ and reaction enthalpy of −461 kJ mol^−1^. Epoxide E via the smallest direct energy barrier TS1 of 207 kJ mol^−1^ dissociated into ketene and methylglyoxal, with an overall reaction enthalpy of −274 kJ mol^−1^. Finally, 5-methyl-2,4-furandione formed through the triplet diradical D intersystem crossing, with a reaction enthalpy of −108 kJ mol^−1^.

## 3. Methods

The experiments were performed at the Chemical Dynamics Beamline 9.0.2 at the Advanced Light Source (ALS) of the Lawrence Berkeley National Laboratory [54]. Reaction species were identified by multiplexed time- and energy-resolved mass spectrometry coupled with tunable synchrotron radiation for photoionization. Details of the apparatus have been provided elsewhere [55,56,57].

AAL (Sigma-Aldrich; purity ≥ 98%) was further purified through the freeze–pump–thaw technique, and its vapors were collected in a gas cylinder to reach 1% mixture with helium. AAL, He, and NO_2_ (photolysis precursor) flowed into the heated slow-flow reaction cell, a 62 cm quartz tube with an inner diameter of 1.05 cm and an outer diameter of 1.27 cm, by calibrated mass flow controllers. Then, the gases effused through a 650 µm wide pinhole on the side of the reactor and flowed into a differentially vacuumed ionization region. The ground-state oxygen atom, O(^3^P), was generated by an unfocused 4 Hz-pulsed 351 nm XeF excimer laser of 1% NO_2_ and helium gas [56]:

NO_2_ → NO + O(^3^P)

At this wavelength, the quantum yield of O(^3^P) is 1.00 (according to Troe) [58], and the absorption cross section is 4.62 × 10^−19^ cm^2^ (according to Vandaele and co-workers) [59]. Based on these values with the concentration of NO_2_ of 1.27 × 10^17^ molecules cm^−3^ at 298 K, 1.17 × 10^17^ molecules cm^−3^ at 550 K, and 9.18 × 10^16^ molecules cm^−3^ at 700 K, and with the laser fluence of 40 mJ cm^−2^ at 298 K and 42 mJ cm^−2^ at 550 and 700 K, the concentration of O(^3^P) was held at 3.81 × 10^15^ molecules cm^−3^ at 298 K, 3.97 × 10^15^ molecules cm^−3^ at 550 K, and 3.10 × 10^15^ molecules cm^−3^ at 700 K.

The ionized species were accelerated and detected by a 50 kHz pulsed orthogonal acceleration time-of-flight mass spectrometer. For these experiments, under the current experimental conditions, the mass resolution was approximately 1600. In this study, AAL reacted with O(^3^P) at different temperatures, i.e., 298, 550, and 700 K. The pressure in the tube was controlled through a closed-loop feedback throttle valve, which was connected to a roots pump. The pressure inside the tube was kept at 4 Torr at 298 K and was increased to 7 Torr at both 550 and 700 K.

A three-dimensional data block, consisting of the ion signal as a function of photon energy (eV), the mass-to-charge ratio (*m*/*z*), and the reaction time (ms), was collected, integrated, and “sliced” into two two-dimensional data plots, reaction time (ms) vs. mass-to-charge ratio (*m*/*z*) and photon energy (eV) vs. mass-to-charge ratio (*m*/*z*). The kinetic time traces were obtained through a vertical slice of 2D images (i.e., the ion signal was integrated for a selected *m*/*z*) of *m*/*z* vs. reaction time, and the photoionization spectra were provided by a vertical slice of 2D images of *m*/*z* vs. photon energy over a specific time range.

The primary products were confirmed by their kinetic time traces, which were superimposed onto the inversed kinetic time trace (i.e., multiplied by −1) of the reactant. Figure 1 is the kinetic time trace of a primary product at *m*/*z* 42 superimposed onto the inverse of AAL at 700 K. A secondary product was disregarded through the analysis when the slope of its kinetic time trace onset was slower than the inverse kinetic time trace of the reactant [56]. In this study, the photoionization spectra were integrated in the 0–40 ms time range at 298 K, 0–30 ms at 550 K, and 0–20 ms at 700 K to minimize the presence of signal due to secondary chemistry, which arises at long times. The primary products were characterized by comparing their photoionization (PI) spectra and adiabatic ionization energies (AIE) with those reported in the literature, measured, or the calculated PI curves. AIE was determined by the linear extrapolation of the initial onset of the photoionization curves [57].

Branching fractions of the primary products can also be derived to quantify the reaction species. Equation (1) shows how the branching fractions are calculated, which correspond to the ratio of the product concentration (C_P_) over that of the reactant (C_R_).

(1) $$\mathrm{Branching}\;\mathrm{Fraction}=\frac{C_{P}}{C_{R}}=\frac{S_{P}\sigma_{R}}{S_{R}\sigma_{P}}\;.\;{\left(\frac{M_{R}}{M_{P}}\right)}^{0.67}$$

S_P_ and S_R_ represent the ion signals at 11 eV in this study. σ_P_ and σ_R_ are the energy-dependent PI cross sections at 11 eV of the product and reactant, respectively. M_R_ and M_P_ are the masses of reactant and primary products, respectively, to the power of 0.67 [60], which takes into account how efficiently the detector can detect a species. The PI cross sections of primary products were obtained from the literature, when available, or were calculated using the semi-empirical additivity method by Bobeldijk [52] when unknown. The uncertainties of the branching fractions were calculated using the propagation of errors of the values in equation 1. The uncertainties of the photoionization cross sections and mass discrimination were obtained from the literature, whereas the ion signal uncertainties were calculated by taking the difference between the measured lower value at 11 eV and its reference signal (from the literature or simulation). The same procedure was used for the upper ion signal value at 11 eV, and the average of two differences was used.

### Computational Methods

When a reaction species is unknown, its possible structures are optimized by electronic structure calculations using the CBS–QB3 (Complete Basis Set) composite model [61,62] with the Gaussian 09 software program [63]. The CBS–QB3 method is used because of its energy accuracy of 4–5 kJ mol^−1^ as given by Montgomery et al. [62]. In addition, this composite model provides reliable structural parameters, bond lengths, bond angles, and harmonic vibrational frequencies, which can be used for spectral simulation [64].

The experimental observable that can be computed is the adiabatic ionization energy (AIE), which provides a first characterization of the studied reaction species. AIE is obtained by taking the difference between the zero-point vibrational corrected total electronic energy (E_0_) of the ground electronic state of the neutral (E_0, neutral_) and the cationic molecule (E_0, cation_) [63], i.e., AIE = E_0, cation_ − E_0, neutral_. The primary products are identified when experimental AIE and overall PI spectra match with the respective reference values. If a literature PI spectrum is not available to verify a specific species, a photoelectron spectrum is simulated using Gaussian 09 for the proposed reaction product. The photoelectron spectrum is generated by the Franck–Condon (FC) and Franck–Condon–Herzberg–Teller methods [65,66,67]. FC overlap integrals are calculated using a recursive formula developed by Ruhoff [68]. The simulated photoelectron spectra are then integrated to yield the simulated PI curves, which can be compared with experimental PI spectra.

After the products are characterized, it is necessary to obtain the reaction pathways leading to the identified primary products. Potential energy surface scans at the B3LYP/6–31G-(d) level of theory are performed as a function of bond lengths and angles to visualize transition state barriers [69]. The energies of transitions states and minima are recalculated using the CBS–QB3 method, and the zero-point-corrected total electronic energies (ZPE) are used. Intrinsic reaction coordination (IRC) calculations are also carried out based on the proposed transition state to verify the forward and reverse steps of the potential energy surface scans, i.e., to confirm that the computed transition state is connecting the two expected local minima [70].

## 4. Conclusions

In this investigation, the oxidation of AAL initiated by O(^3^P) was studied at 298, 550, and 700 K. This reaction was carried out at the Lawrence Berkeley National Laboratory using synchrotron radiation coupled with a multiplex photoionization mass spectrometer. The primary products were characterized by mass-to-charge ratios, adiabatic ionization energies, and photoionization spectra. The only observed pathway was the O(^3^P) addition to the unsaturated carbons. The hydrogen abstraction pathway by atomic oxygen was not kinetically favorable because it proceeds through a high energy barrier. The O(^3^P) addition pathway started with the formation of two triplet diradicals (C and D), which underwent intersystem crossing to form the singlet epoxide (E). The reaction pathways were computed using the CBS–QB3 composite model. Four products were generated by unimolecular dissociation of epoxide E, ketene, acetaldehyde, methyl vinyl ketone, and methylglyoxal. Two products formed directly from the diradical D via intersystem crossing, i.e., dimethyl glyoxal and 5-methyl-2,4-furandione. The branching fraction of each primary product was C atom-balanced, i.e., for our reactant AAL, the total branching fractions summed to 500% because of the presence of five carbon atoms. Therefore, each single branching fraction was multiplied by the C atom number present in the specific product and divided by 5 to report the total branching fractions to 100%. The most abundant product at all temperatures was ketene, (47 ± 15)% at 298 K, (40 ± 13)% at 550 K, and (40 ± 12)% at 700 K, followed by methyl vinyl ketone (35 ± 13)% at 298 K, (27 ± 11)% at 550 K, and (20 ± 8)% at 700 K. Methylglyoxal was the third most abundant product at 298 K, (11 ± 4)%, whereas acetaldehyde was the third most abundant product at both 550 K, (9 ± 4)% and 700 K, (16 ± 7)%.

## Figures and Tables

**Table 1:** C atom-balanced branching fraction of products with their uncertainties observed through the oxidation of AAL initiated by O(^3^P) at 298, 550, and 700 K.

Compound	*m*/*z*	298 K	550 K	700 K
ketene	42	47.2 ± 14.6	39.5 ± 13.4	39.7 ± 12.2
acetaldehyde	44	5.6 ± 2.1	9.3 ± 4.2	15.9 ± 7.1
methyl vinyl ketone	70	34.6 ± 12.6	26.7 ± 10.7	19.7 ± 7.6
methylglyoxal	72	11.1 ± 3.9	7.4 ± 3.6	4.7 ± 2.2
dimethylglyoxal	86	-	3.5 ± 1.4	3.4 ± 2.0
5-methyl-2,4-furandione	114	2.2 ± 1.5	1.0 ± 0.5	-
**Total** (C balanced)		101 ± 19.9	87.3 ± 18.1	83.4 ± 16.3

## Data Availability

The data presented in this study are available on request from the corresponding author.

## References

[1] Ranzan L., Ranzan C., Trierweiler L.F., Trierweiler J.O. (2017). Classification of Diesel Fuel Using Two-Dimensional Fluorescence Spectroscopy. Energy Fuels.

[2] Ramanathan V., Feng Y. (2009). Air pollution, greenhouse gases and climate change: Global and regional perspectives. Atmos. Environ..

[3] Chang T., Zivin J.S.G., Gross T., Neidell M. (2014). Particulate Pollution and the Productivity of Pear Packers.

[4] Kampa M., Castanas E. (2008). Human health effects of air pollution. Environ. Pollut..

[5] Atsumi S., Hanai T., Liao J.C. (2008). Non-Fermentative Pathways for Synthesis of Branched-Chain Higher Alcohols as Biofuels. Nature.

[6] Hill J., Nelson E., Tilman D., Polasky S., Tiffany D. (2006). Environmental, Economic, and Energetic Costs and Benefits of Biodiesel and Ethanol Biofuels. Proc. Natl. Acad. Sci. USA.

[7] Kohse-Höinghaus K., Osswald P., Cool T.A., Kasper T., Hansen N., Qi F., Westbrook C.K., Westmoreland P.R. (2010). Biofuel Combustion Chemistry: From Ethanol to Biodiesel. Angew. Chem. Int. Ed..

[8] Vispute T.P., Huber G.W. (2008). Breaking the Chemical and Engineering Barriers to Lignocellulosic Biofuels. Int. Sugar J..

[9] Malpani S., Joseph E. (2015). 2,5-Dimeethylfuran as a Bio-Fuel. J. Environ. Sci. Toxicol. Food Technol..

[10] Mascal M., Nikitin E.B. (2008). Direct, High-Yield Conversion of Cellulose into Biofuel. Angew. Chem. Int. Ed..

[11] Escobar J.C., Lora E.S., Venturini O.J., Yáñez E.E., Castillo E.F., Almazan O. (2009). Biofuels: Environment, technology and food security. Renew. Sustain. Energy Rev..

[12] Fathi Y., Meloni G. (2017). Study of the Synchrotron Photoionization Oxidation of 2-Methylfuran Initiated by O(^3^P) under Low-Temperature Conditions at 550 and 650 K. J. Phys. Chem. A.

[13] Ritter S. (2011). Race To The Pump. Chem. Eng. News Arch..

[14] Sims R., Taylor M., Saddler J., Mabee W. (2008). From 1st to 2nd-generation biofuel technologies: An overview of current industry and RD&D activities. Int. Energy Agen..

[15] Binod P., Gnansounou E., Sindhu R., Pandey A. (2019). Enzymes for second generation biofuels: Recent developments and future perspectives. Bioresour. Technol. Rep..

[16] Zhang Y., Bi P., Wang J., Jiang P., Wu X., Xue H., Liu J., Zhou X., Li Q. (2015). Production of jet and diesel biofuels from renewable lignocellulosic biomass. Appl. Energy.

[17] Avelino F., Silva K.T., de Souza Filho M.d.S.M., Mazzetto S.E., Lomonaco D. (2018). Microwave-assisted organosolv extraction of coconut shell lignin by Brønsted and Lewis acids catalysts. J. Clean. Prod..

[18] Hronec M., Fulajtárova K., Liptaj T., Štolcová M., Prónayová N., Soták T. (2014). Cyclopentanone: A raw material for production of C15 and C17 fuel precursors. Biomass Bioenergy.

[19] Zaras A.M., Dagaut P., Serinyel Z. (2014). Computational Kinetic Study for the Unimolecular Decomposition Pathways of Cyclohexanone. J. Phys. Chem. A.

[20] Yang J., Li N., Li G., Wang W., Wang A., Wang X., Cong Y., Zhang T. (2014). Synthesis of renewable high-density fuels using cyclopentanone derived from lignocellulose. Chem. Commun..

[21] Otera J. (1993). Transesterification. Chem. Rev..

[22] Hoover S.W., Marner W.D., Brownson A.K., Lennen R.M., Wittkopp T.M., Yoshitani J., Zulkifly S., Graham L.E., Chaston S.D., McMahon K.D. (2011). Bacterial Production of Free Fatty Acids from Freshwater Macroalgal Cellulose. Appl. Microbiol. Biotechnol..

[23] Park E.Y., Sato M., Kojima S. (2006). Fatty Acid Methyl Ester Production Using Lipase-Immobilizing Silica Particles with Different Particle Sizes and Different Specific Surface Areas. Enzym. Microb. Tech..

[24] Schuchardt U., Sercheli R., Vargas R.M. (1998). Transesterification of Vegetable Oils: A Review. J. Braz. Chem. Soc..

[25] Balakrishnan M., Sacia E.R., Bell A.T. (2012). Etherification and Reductive Etherification of 5-(Hydroxymethyl)Furfural: 5-(Alkoxymethyl)Furfurals and 2,5-Bis(Alkoxymethyl)Furans as Potential Bio-Diesel Candidates. Green Chem..

[26] Frusteri F., Frusteri L., Cannilla C., Bonura G. (2012). Catalytic Etherification of Glycerol to Produce Biofuels over Novel Spherical Silica Supported Hyflon Catalysts. Bioresour. Technol..

[27] Agarwal A.K. (2007). Biofuels (Alcohols and Biodiesel) Applications as Fuels for Internal Combustion Engines. Prog. Energy Combust. Sci..

[28] Rodriguez G.M., Atsumi S. (2012). Synthetic Biology Approaches to Produce C3-C6 Alcohols from Microorganisms. Curr. Chem. Biol..

[29] Román-Leshkov Y., Barrett C.J., Liu Z.Y., Dumesic J.A. (2007). Production of Dimethylfuran for Liquid Fuels from Biomass-Derived Carbohydrates. Nature.

[30] Mehdi H., Fábos V., Tuba R., Bodor A., Mika L.T., Horváth I.T. (2008). Integration of Homogeneous and Heterogeneous Catalytic Processes for a Multi-step Conversion of Biomass: From Sucrose to Levulinic Acid, γ-Valerolactone, 1,4-Pentanediol, 2-Methyl-tetrahydrofuran, and Alkanes. Top. Catal..

[31] Horváth I.T., Mehdi H., Fábos V., Boda L., Mika L.T. (2008). γ-Valerolactone—A Sustainable Liquid for Energy and Carbon-Based Chemicals. Green Chem..

[32] Bond J.Q., Alonso D.M., Wang D., West R.M., Dumesic J.A. (2010). Integrated Catalytic Conversion of-Valerolactone to Liquid Alkenes for Transportation Fuels. Science.

[33] Cotton S. (2008). Lactones as Biofuel. https://edu.rsc.org/soundbite/lactones-as-biofuel/2021245.article.

[34] Simmie J.M. (2003). Detailed Chemical Kinetic Models for the Combustion of Hydrocarbon Fuels. Prog. Energy Combust. Sci..

[35] Welz O., Zádor J., Savee J.D., Ng M.Y., Meloni G., Fernandes R.X., Sheps L., Simmons B.A., Lee T.S., Osborn D.L. (2012). Low-Temperature Combustion Chemistry of Biofuels: Pathways in the Initial Low-Temperature (550 K–750 K) Oxidation Chemistry of Isopentanol. Phys. Chem. Chem. Phys..

[36] Giustini A., Meloni G. (2020). Synchrotron Photoionization Study of the Diisopropyl Ether Oxidation. ChemPhysChem.

[37] Shi Y., Ge H.-W., Brakora J.L., Reitz R.D. (2010). Automatic Chemistry Mechanism Reduction of Hydrocarbon Fuels for HCCI Engines Based on DRGEP and PCA Methods with Error Control. Energy Fuels.

[38] Cool T.A., Nakajima K., Mostefaoui T.A., Qi F., McIlroy A., Westmoreland P.R., Law M.E., Poisson L., Peterka D.S., Ahmed M. (2003). Selective Detection of Isomers with Photoionization Mass Spectrometry for Studies of Hydrocarbon Flame Chemistry. J. Chem. Phys..

[39] Zádor J., Taatjes C.A., Fernandes R.X. (2011). Kinetics of Elementary Reactions in Low-Temperature Autoignition Chemistry. Prog. Energy Combust. Sci..

[40] Lima C.G., Monteiro J.L., de Melo Lima T., Weber Paixão M., Corrêa A.G. (2017). Angelica Lactones: From Biomass-Derived Platform Chemicals to Value-Added Products. ChemSusChem.

[41] McAdam K., Enos T., Goss C., Kimpton H., Faizi A., Edwards S., Wright C., Porter A., Rodu B. (2018). Analysis of Coumarin and Angelica Lactones in Smokeless Tobacco Products. Chem. Cent. J..

[42] Cao R., Xin J., Zhang Z., Liu Z., Lu X., Ren B., Zhang S. (2014). Efficient Conversion of α-Angelica Lactone into γ-Valerolactone with Lonic Liquids at Room Temperature. ACS Sustain. Chem. Eng..

[43] Valco D.J., Min K., Oldani A., Edwards T., Lee T. (2017). Low temperature autoignition of conventional jet fuels and surrogate jet fuels with targeted properties in a rapid compression machine. Proc. Combust. Inst..

[44] Cvetanović R.J. (1987). Evaluated Chemical Kinetic Data for the Reactions of Atomic Oxygen O(^3^P) with Unsaturated Hydrocarbons”. J. Phys. Chem. Ref. Data.

[45] González-Lavado E., Corchado J.C., Espinosa-Garcia J. (2014). The Hydrogen Abstraction Reaction O(^3^P) + CH_4_: A New Analytical Potential Energy Surface Based on Fit to Ab Initio Calculations. J. Chem. Phys..

[46] Watanabe K., Matsunaga F.M., Sakai H. (1967). Absorption Coefficient and Photoionization Yield of NO in the Region 580–1350 Å. Appl. Opt..

[47] Goulay F., Derakhshan A., Maher E., Trevitt A.J., Savee J.D., Scheer A.M., Osborn D.L., Taatjes C.A. (2013). Formation of Dimethylketene and Methacrolein by Reaction of the CH Radical with Acetone. Phys. Chem. Chem. Phys..

[48] Yang B., Wang J., Cool T.A., Hansen N., Skeen S., Osborn D.L. (2012). Absolute Photoionization Cross-Sections of Some Combustion Intermediates. Int. J. Mass Spectrom..

[49] Reed R.I., Brand J.C. (1958). Electron Impact Studies. Part 4—Glyoxal, Methylglyoxal, and Diacetyl. Trans. Faraday Soc..

[50] Thorstad O., Undheim K., Cederlund B., Hörnfeldt A.-B., Servin R., Sternerup H. (1975). Ionisation Potentials in Tautomeric Analysis of 2-Hydroxy Derivatives of Thiophenes, Selenophenes, and Furans. Acta Chem. Scand..

[51] Czekner J., Taatjes C.A., Osborn D.L., Meloni G. (2013). Absolute Photoionization Cross-Sections of Selected Furanic and Lactonic Potential Biofuels. Int. J. Mass Spectrom..

[52] Bobeldijk M., van der Zande W.J., Kistemaker P.G. (1994). Simple Models for the Calculation of Photoionization and Electron Impact Ionization Cross Sections of Polyatomic Molecules. Chem. Physics.

[53] Cool T.A., Wang J., Nakajima K., Taatjes C.A., Mcllroy A. (2005). Photoionization Cross Sections for Reaction Intermediates in Hydrocarbon Combustion. Int. J. Mass Spectrom..

[54] Heimann P.A., Koike M., Hsu C.W., Blank D., Yang X.M., Suits A.G., Lee Y.T., Evans M., Ng C.Y., Flaim C. (1997). Performance of the Vacuum Ultraviolet High-Resolution and High-Flux Beamline for Chemical Dynamics Studies at the Advanced Light Source. Rev. Sci. Instrum..

[55] Fathi Y., Price C., Meloni G. (2017). Low-Temperature Synchrotron Photoionization Study of 2-Methyl-3-buten-2-ol (MBO) Oxidation Initiated by O(^3^P) Atoms in the 298–650 K Range. J. Phys. Chem. A.

[56] Ray A.W., Taatjes C.A., Welz O., Osborn D.L., Meloni G. (2012). Synchrotron Photoionization Measurements of OH-Initiated Cyclohexene Oxidation: Ring-Preserving Products in OH^+^ Cyclohexene and Hydroxycyclohexyl + O_2_ Reactions. J. Phys. Chem. A.

[57] Ng M.Y., Bryan B.M., Nelson J., Meloni G. (2015). Study of tert-Amyl Methyl Ether Low-Temperature Oxidation Using Synchrotron Photoionization Mass Spectrometry. J. Phys. Chem. A.

[58] Troe J. (2000). Are Primary Quantum Yields of NO_2_ Photolysis at λ ≤ 398 nm Smaller Than Unity?. Z. Phys. Chem..

[59] Vandaele A.C., Hermans C., Simon P.C., Carleer M., Colin R., Fally S., Mérienne M.F., Jenouvrier A., Coquart B. (1998). Measurements of the NO_2_ Absorption Cross-Section from 42 000 cm^−1^ to 10 000 cm^−1^ (238–1000 nm) at 220 K and 294 K. J. Quant. Spectrosc. Radiat. Transf..

[60] Savee J.D., Soorkia S., Welz O., Selby T.M., Taatjes C.A., Osborn D.L. (2012). Absolute Photoionization Cross-Section of the Propargyl Radical. J. Chem. Phys..

[61] Montgomery J.A., Frisch M.J., Ochterski J.W., Petersson G.A. (1999). A Complete Basis Set Model Chemistry. VI. Use of Density Functional Geometries and Frequencies. J. Chem. Phys..

[62] Montgomery J.A., Frisch M.J., Ochterski J.W., Petersson G.A. (2000). A Complete Basis Set Model Chemistry. VII. Use of the Minimum Population Localization Method. J. Chem. Phys..

[63] Frisch M.J., Trucks G.W., Schlegel H.B., Scuseria G.E., Robb M.A., Cheeseman J.R., Scalmani G., Barone V., Mennucci B., Petersson A. (2009). Gaussian 09.

[64] Winfough M., Yao R., Ng M., Catani K., Meloni G. (2017). Synchrotron Photoionization Investigation of the Oxidation of Ethyl tert-Butyl Ether. J. Phys. Chem. A.

[65] Duschinsky F. (1937). The importance of the electron spectrum in multi atomic molecules. Concerning the Franck-Condon principle. Acta Physicochim URSS.

[66] Sharp T.E., Rosenstock H.M. (1964). Franck—Condon Factors for Polyatomic Molecules. J. Chem. Phys..

[67] Lermé J. (1990). Iterative Methods to Compute One- and Two-Dimensional Franck-Condon Factors. Tests of Accuracy and Application to Study Indirect Molecular Transitions. Chem. Phys..

[68] Ruhoff P.T. (1994). Recursion Relations for Multi-Dimensional Franck-Condon Overlap Integrals. Chem. Phys..

[69] Tirado-Rives J., Jorgensen W.L. (2008). Performance of B3LYP Density Functional Methods for a Large Set of Organic Molecules. J. Chem. Theory Comput..

[70] Fukui K. (1981). The Path of Chemical Reactions—the IRC Approach. Acc. Chem. Res..

